# Development and validation of a predictive nomogram for severe adverse drug reactions: a dual-center pharmacovigilance study

**DOI:** 10.3389/fphar.2025.1669995

**Published:** 2025-11-07

**Authors:** Wei Bu, Xinjing Wu, Chengyu Wang, Yan Cai

**Affiliations:** 1 Department of Pharmacy, Affiliated Hospital of Zunyi Medical University, Zunyi, Guizhou, China; 2 Department of Pharmacy, Yuncheng Central Hospital Affiliated to Shanxi Medical University, Yuncheng, Shanxi, China; 3 Department of Pharmacy, The Second Affiliated Hospital of Xi’an Jiaotong University, Xi’an, Shaanxi, China

**Keywords:** severe adverse drug reactions, adverse drug reactions, machine learning, nomogram, predictive model

## Abstract

**Background:**

Severe adverse drug reactions (SADRs) pose significant challenges to pharmacotherapy. Machine learning (ML) models hold promise in providing reliable solutions for predicting SADRs. This study is designed to pinpoint the independent risk factors contributing to SADRs through the application of ML techniques, thus constructing a predictive model for SADRs applicable in real-world clinical settings.

**Methods:**

This retrospective dual-center cohort study analyzed adverse drug reaction (ADR) cases reported in two Chinese tertiary medical centers from 2014 to 2022. Per the World Health Organization - Uppsala Monitoring Centre severity criteria, cases were classified as SADRs or common ADRs. Independent predictors were identified via univariate and multivariate logistic regression (LR). A random partitioning of the data set resulted in a 75% training set and a 25% test set. The performance of three ML algorithms, including LR, Random Forest and Gradient Boosting Machine, was compared. A nomogram was constructed, model performance was measured by the area under the receiver operating characteristic curve (AUC), concordance index (C index), Hosmer-Lemeshow test (H-L test), Decision Curve Analysis (DCA), and Clinical Impact Curve (CIC).

**Results:**

A total of 508 SADRs were identified. The AUC values of LR model demonstrates the highest predictability among the three ML models. The AUC was 0.707 in the test set and the AUC in the training set was 0.689. A nomogram was established based on the LR model and evaluated. The C-index was 0.714 in the test set and the AUC in the training set was 0.713; The H-L test produced a chi-square value of 9.769 (*p* = 0.369), indicating good calibration. The DCA and CIC verify that the LR model possesses significant predictive value. According to the LR model, there were 20 predictors, including age ≥54 years, concurrent diseases ≥3, cardiac insufficiency, hemorrhagic disorders, active malignancies, cerebral infarction, bone fractures, anti-infectives, cytotoxic antineoplastics, proton pump inhibitors, antiepileptics, anticoagulants, diagnostic agents, arterial administration.

**Conclusion:**

This study established a predictive nomogram for SADRs based on LR through comparative analysis of three ML approaches. The developed nomogram enables clinically meaningful risk stratification for SADRs, facilitating prophylactic surveillance of high-risk populations.

## Introduction

Adverse drug reactions (ADRs) refer to harmful and unexpected pharmacological reactions that manifest at standard therapeutic doses (Montané and Santesmases, 2020). Approximately 10%–20% of inpatients and 25% of outpatients experience ADRs (Garon et al., 2017). Systematic review and meta - analysis reveal marked variations in the incidence of serious adverse drug reactions (SADRs) linked to hospitalization, spanning from 1.0% to 16.8% (Formica et al., 2018). It was showed that 6.7% of hospitalized patients incur SADRs, with a 0.32% prevalence of fatal ADRs (Formica et al., 2018). Alarmingly, research in pharmacovigilance has shown that ADRs are the fourth top cause of death around the world (Moyer et al., 2019).

Pharmacovigilance constitutes a pivotal strategy for the prevention and mitigation of adverse drug events (ADEs) and ADRs (Song et al., 2023). Methodologically, it is dichotomised into passive and active monitoring systems (Li and Yin, 2019). Passive surveillance relies on spontaneous reporting systems (SRS), through which healthcare professionals voluntarily submit observed ADRs. Owing to its minimal infrastructural requirements, SRS is the predominant mode of data collection in many jurisdictions (Mulchandani and Kakkar, 2019). Its advantages include high data volume, ease of access, and low operational cost. Nevertheless, this approach is intrinsically limited by under-reporting, duplication, low reporting rates, and variable data quality (Shamim et al., 2024; Crisafulli et al., 2025; Alatawi and Hansen, 2017). Active pharmacovigilance leverages comprehensive electronic health records that encapsulate detailed patient-level information, thereby enabling the simultaneous control of confounders such as polypharmacy, combination therapies, and sociodemographic characteristics (Zhuo et al., 2014). However, the substantial human and financial resources required for its implementation have constrained its widespread adoption. Recently, artificial intelligence (AI), particularly machine learning (ML), has emerged as a transformative paradigm in pharmacovigilance (Demirsoy and Karaibrahimoglu, 2023; Hu et al., 2024; Salas et al., 2022). By learning patterns from large-scale data, these algorithms facilitate the automated identification of ADEs/ADRs, extraction of drug–drug interactions, and stratification of patients at elevated ADR risk (Salas et al., 2022). The principal advantages of AI-driven approaches are threefold: (i) Automated report generation and multimodal data integration, amalgamating electronic medical records, genomic data, social media content, and other heterogeneous sources, thereby enhancing both the velocity and accuracy of data processing (Kompa et al., 2022; Golder et al., 2025; Dsouza et al., 2025; Dimitsaki et al., 2024). (ii) Objective risk assessment, wherein AI models generate causal probability scores by systematically analysing confounding factors, temporal relationships, and the hierarchy of literature evidence, thereby mitigating subjective bias (Desai, 2024). (iii) Individualised risk prediction, achieved through predictive models that integrate patients’ genomic, metabolic, and medication histories to deliver personalised ADR risk estimates (Ward et al., 2021). Although ML approaches have demonstrated significant advantages in ADR research, the prediction of ADRs still face challenges, particularly as studies on ML models for predicting SADRs remain scarce.

Given the significant adverse impact of SADRs on patient health, it is important to identify risk factors associated with SADRs and develop predictive models. This retrospective study aims to establish an accurate and reliable model for predicting SADRs by using ML algorithms.

## Materials and methods

### Study design

Pharmacovigilance data from two different tertiary care institutions in China were used in this dual-center retrospective cohort study: Affiliated Hospital of Zunyi Medical University (Zunyi, Guizhou); Yuncheng Central Hospital affiliated to Shanxi Medical University (Yuncheng, Shanxi). The dataset spans from 1 January 2014, to 31 December 2022, and includes all ADR reports registered in the national surveillance systems of the institutions involved.

This study was conducted in accordance with the Declaration of Helsinki and approved by the Ethics Committee of the Affiliated Hospital of Zunyi Medical University (Approval No. KLL-2021-257) and the collaborating center. The requirement for written informed consent was waived by the Ethics Committee due to the retrospective nature of the research.

### Data assessment

Experts from the Drug Reevaluation Centre of China National Medical Products Administration evaluated all the adverse drug reactions (ADRs), which were categorized as “certain”, “probable”, “possible”, “irrelevant”, “to be evaluated”and “unable to evaluate” based on World Health Organization - Uppsala Monitoring Centre (WHO-UMC) causality assessment criteria (Chen et al., 2018). The authors reassessed the causal relationship of the ADR using the Naranjo algorithm (Shukla et al., 2021). Inclusion criteria were limited to ADRs categorized as “certain”, “probable”, or “possible” (The Uppsala Monitoring Centre, 2013). Cases were excluded if: (i) Causality assessments classified as “irrelevant”, “to be evaluated” or “unable to evaluate”; (ii) Key information were missing, as gender, age; (iii) Occurrence time was unclear; (iv) Diagnosis incomplete.

In accordance with ICH E2A guidelines (Therapeutic Goods Administration of Department of Health of Australian Government, 2000), SADRs are defined as any event meeting at least one of the following standards: (i) Requires inpatient hospitalization or prolongation of hospitalization; (ii) Results in persistent or significant disability/incapacity; (iii) A congenital anomaly/birth defect; (iv) Life-threatening; (v) Fatal outcome; (vi) Other serious medical events that may lead to the aforementioned outcomes (Chen et al., 2018).

Additionally, ADR outcomes were typically encompassed within several definitive categories: cured, improvement, recovered with sequelae, no healing, death and unknown (Sun et al., 2020).

The MedDRA v24.0 classification system was used to describe organ-specific injury manifestations of common ADRs and SADRs (International Council for Harmonisation of Technical Requirements for Pharmaceuticals for Human Use, 2021). The WHO-ATC classification system was employed to evaluate the ADR profiles of different drug categories (Norwegian Institute of Public Health, 2025).

### Data analysis

SPSS (version 26.0, Chicago, IL, United States) and R software (version 3.5.1, R Foundation for Statistical Computing, Vienna, Austria) were used for statistical analyses. The Shapiro test was employed to evaluate the distribution of continuous variables. For normallydistributed variables, data were shown as mean ± standard deviation (SD), while skewed data were presented as median (interquartile range, IQR). Categorical data were represented by frequencies and percentages.

The comparison of the common ADRs cohort with the SADRs cohort was conducted using the unpaired Student’s t-test or the non-parametric Mann-Whitney test for continuous variables, and chi-square or Fisher’s exact tests for categorical variables. Through receiver operating characteristic (ROC) curve analysis, continuous variables that showed statistical significance were divided into two groups, with the Youden index used to find the optimal diagnostic thresholds (Obuchowski and Bullen, 2018). To identify risk factors for SADRs, chi-square (or Fisher’s exact) tests or univariate LR analysis were conducted. In the univariate analysis, variables with p-values <0.10 along with clinically relevant parameters were included in the multivariate LR model via forward stepwise selection (Faria et al., 2022; Eastment et al., 2019). Clinically relevant parameters were included in the multivariate LR model via forward stepwise selection (Faria et al., 2022). Two-tailed tests were used, and a *P* value below 0.05 was regarded as statistically significant.

In this study, a fixed ratio of 75%-25% was employed for data partitioning in accordance with the requirements of TRIPOD-AI and PROBAST by using the R language (via the createDataPartition function from the caret package).

In building the model, three ML algorithms were used: LR, Random Forest (RF), and Gradient Boosting Machines (GBM). The hyperparameter tuning part is in the Supplementary File 1. These classifiers were systematically implemented to construct prediction systems using two independent datasets containing optimally selected feature variables (Lin et al., 2024). The effectiveness of each model’s predictions was assessed using the area under the receiver operating characteristic curve (AUC) (Li et al., 2022). Furthermore, we also calculated specificity, accuracy, sensitivity, F1-score and precision for each model’s AUC at the “best” thresholds (Zhang S. T. et al., 2025; Peng et al., 2025). Bootstrap calculated using 1,000 stratified repetitions in the “pROC” program.

### Nomogram implementation and test

Using the “rms” package in R statistical software, a nomogram for SADRs was developed from a multivariate LR model (Hoshino et al., 2018). Its performance was assessed with a concordance index (C-index) and calibration plots derived from bootstrap samples. Calibration plots provide a visual evaluation of predictive accuracy by comparing observed probabilities with those predicted by the nomogram, while a C-index measures discriminative ability numericallya. Decision Curve Analysis (DCA) was utilized to measure the clinical utility of the predictive models. By assessing the net benefit over various threshold probabilities, DCA allows for comparison of the nomogram with other models and highlights their respective differences. By showing the false-positive and true-positive rates as functions of the risk threshold, DCA effectively addresses the limitations of ROC curves (Zhang et al., 2022). Finally, the net clinical benefit of the model with the best diagnostic results was assessed by plotting clinical impact curves (CIC) (Hou et al., 2020).

## Results

### Baseline characteristics

**FIGURE 1:**
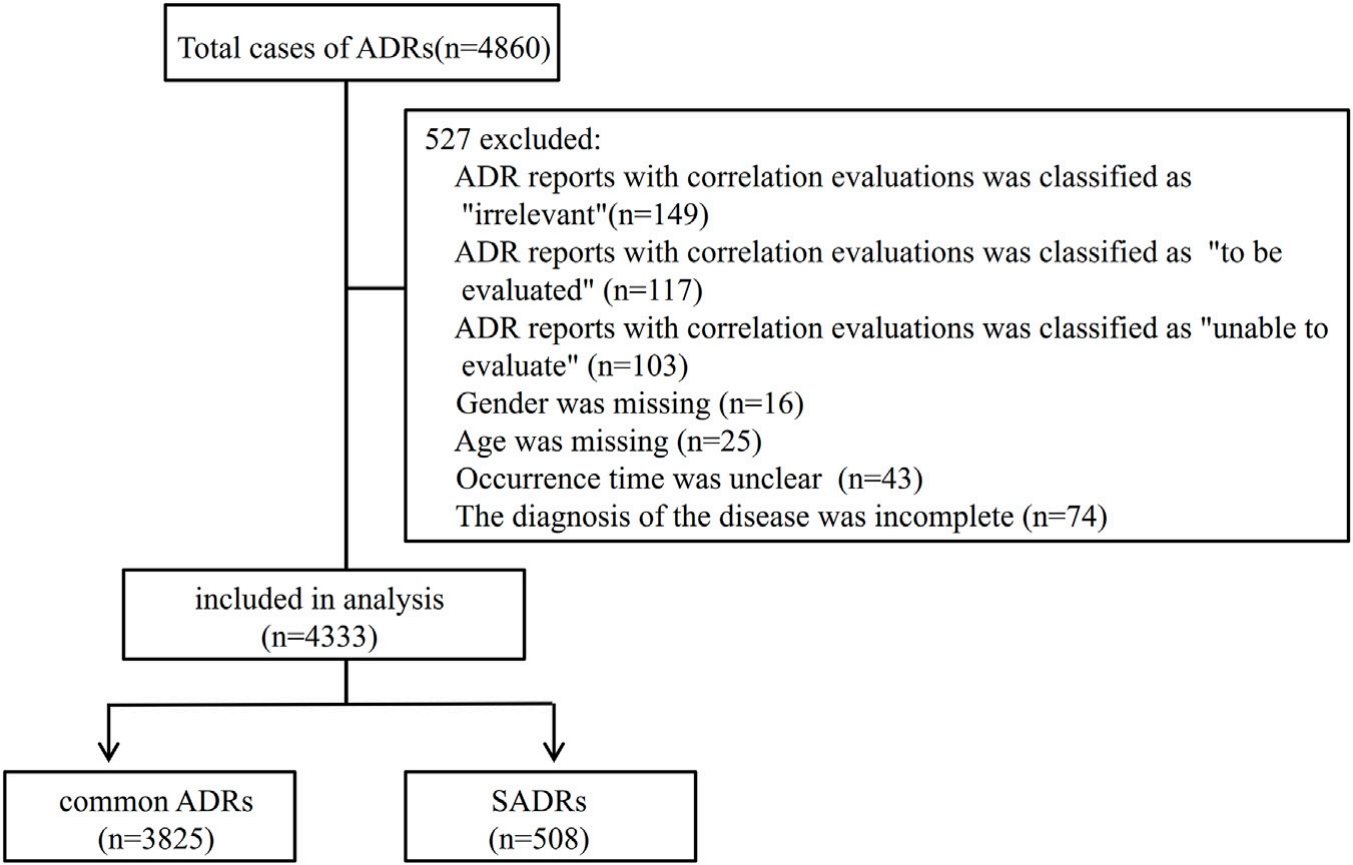
ADRs selection. SADRs: serious adverse drug reactions.

Of the 4,860 cases, 4,333, accounting for 89.2%, fulfilled the inclusion/exclusion criteria and were retained for subsequent analysis (Figure 1). Causality assessment using the WHO-UMC criteria classified 2.5% (n = 109) of the cases as “certain,” with the remainder distributed between “probable” (62.4%, n = 2,702) and “possible” (35.1%, n = 1,522).

To better understand the risk factors associated with SADRs, the cases were divided into two cohorts: the common ADRs cohort (3,825 cases, 88.3%) and the SADRs cohort (508 cases, 11.7%). A comparison of demographic and clinical characteristics is shown in Table 1. The key findings are summarized as follows: (i) Male patients exhibited higher risks of SADRs, constituting 53.3% of SADRs. (ii) Patients aged 54 years and older had a significantly higher proportion of SADRs compared to those younger than 54 years (58.3% vs. 41.7%, *p* < 0.001). (iii) Among the routes examined, IA administration was associated with high occurrences of SADRs (2.6% vs. 0.3% for common ADRs). (iv) There were significant differences (*p* < 0.001) between SADRs and common ADRs in ADR type, clinical aoutcomes, impacts on the primary disease, and the occurrence time of ADRs.

**TABLE 1:** Baseline characteristics of 4,333 ADRs.

Characteristics		All ADRs (4,333)	Common ADRs (3,825)	Severe ADRs (508)	*t/X^2^/Z*	*P* value
Male		2,119 (48.9%)	1,848 (48.3%)	271 (53.3%)	4.546	**0.033**
Age ≥54 years		2,076 (47.9%)	1,780 (46.5%)	296 (58.3%)	24.732	**<0.001**
Previous ADR history	Yes	175 (4.0%)	153 (4.0%)	22 (4.3%)	0.242	0.885
	No	2,263 (52.2%)	2,002 (52.3%)	261 (51.4%)		
	Missing	1895 (43.7%)	1,670 (43.7%)	225 (44.3%)		
Administration routes	IV	2,822 (65.1%)	2,483 (64.9%)	339 (66.7%)	27.879	**<0.001**
	PO	1,204 (27.8%)	1,082 (28.3%)	126 (24.8%)		
	SC	117 (2.7%)	103 (2.7%)	14 (2.8%)		
	IA	25 (0.6%)	12 (0.3%)	13 (2.6%)		
	Others	165 (3.8%)	145 (3.8%)	16 (3.1%)		
Types of ADR	Old	4,018 (92.7%)	3,525 (92.2%)	493 (97.0%)	15.909	**<0.001**
	New	315 (7.3%)	300 (7.8%)	15 (3.0%)		
Outcomes of ADR	Cured	1,458 (33.6%)	1,290 (33.7)	168 (33.1)	35.844	**<0.001**
	Improvement	2,508 (57.9%)	2,202 (57.6)	306 (60.2)		
	Recovered with sequelae	0 (0.0%)	0 (0.0%)	0 (0.0%)		
	No healing	46 (1.1%)	30 (0.8%)	16 (3.1%)		
	Death	0 (0.0%)	0 (0.0%)	0 (0.0%)		
	Unknown	321 (7.4%)	303 (7.9)	18 (3.5)		
Impacts on primary disease	Unobvious	4,013 (92.6%)	3,663 (95.8%)	350 (68.9%)	333.094	**<0.001**
	Prolonged course of primary disease	278 (6.4%)	150 (3.9%)	128 (25.2%)		
	Aggravation	38 (0.9%)	8 (0.2%)	30 (5.9%)		
	Unknown	4 (0.1%)	4 (0.1%)	0 (0.0%)		
Causality assessments	Probable	2,702 (62.4%)	2,481 (64.9%)	221 (43.5%)	99.030	**<0.001**
	Possible	1,522 (35.1%)	1,243 (32.5%)	279 (54.9%)		
	Certain	109 (2.5%)	101 (2.6%)	8 (1.6%)		
Occurrence time	≤5 min	180 (4.2%)	149 (3.9%)	31 (6.1%)	32.164	**<0.001**
	6–30 min	531 (12.3%)	467 (12.2%)	64 (12.6%)		
	31–60 min	745 (17.2%)	676 (17.7%)	69 (13.6%)		
	1 h–24 h	894 (20.6%)	771 (20.2%)	123 (24.2%)		
	1 d–7 d	1650 (38.1%)	1489 (38.9%)	161 (31.7%)		
	8 days–30 days	333 (7.7%)	273 (7.1%)	60 (11.8%)		

Abbreviation: Iv: Intravenous administration (including intravenous drip and intravenous injection); Po: Oral administration; Sc: Subcutaneous injection; IA: Intra-arterial injection; Other administration routes include: 48 cases of inhalation (2 cases of SADRs), 59 cases of intramuscular (7 cases of SADRs), 22 cases of local topical application (3 cases of SADRs), 12 cases of nasal feeding (0 cases of SADRs), and 24 cases of other administration routes (4 cases of SADRs). Bolded values denote statistically significant differences.

Compared with common ADRs, SADRs showed significantly higher incidence in systemic organ injury, hepatobiliary dysfunction, hematologic abnormalities and urinary system injury (*p* < 0.05) (Supplementary File 2; Supplementary Tables S1, S2).

In terms of medications, diagnostic drugs had the highest proportion in SADRs at 28.9%, followed by central nervous system agents (18.0%), hematopoietic modulators (16.9%), anti-tumor medications (15.5%), gastrointestinal drugs (12.6%), immunomodulatory compounds (12.2%), anti-infective agents (12.0%), and traditional Chinese medicine preparations (11.0%) (Supplementary File 2; Supplementary Tables S3).

### Univariate analysis of SADRs

Univariate analysis of the association between patients’ diseases and the occurrence of SADRs are shown in Supplementary File 2; Supplementary Table S4, which yielded the following results: (i) multiple comorbidities: patients with concurrent diseases ≥3 had elevated risks of SADRs compared to those with fewer than 3 concurrent diseases (*p* < 0.001). (ii) Disease-specific risks (*p* < 0.05): active malignancies, cardiac insufficiency, hemorrhagic disorder, cerebral infarction and bone fractures.

The univariate analysis results showed no statistically significant association between multidrug combination therapy and SADRs incidence (*p* > 0.05). The subsequent univariate LR analysis of SADRs included drugs that met the inclusion criteria of having 10 or more recorded SADR cases and an increased incidence. The results showed that SADRs occurrence were significantly associated with the following drugs (*p* < 0.05): ceftazidime, ceftriaxone, cefoperazone/sulbactam, carbapenems, vancomycin, antifungal agents, antiviral medications, antiepileptics, cytotoxic antineoplastics, antithrombotic agents, proton pump inhibitors and diagnostic agents (Supplementary File 2; Supplementary Table S5).

### Multivariate analysis of SADRs

The “administration routes” were dichotomized into intra-arterial (IA) versus non-IA administration. All predictors were assessed for multicollinearity, with only those exhibiting a variance inflation factor <10 being retained in the model (Supplementary File 2; Supplementary Table S6). At last, the model identified 20 independent predictors of SADRs (Supplementary File 2; Supplementary Tables S7): (i) demographic predictors included age ≥54 years (OR 1.280, 95% CI 1.041–1.573; *p* = 0.019) and multi-morbidity (concurrent diseases ≥3: OR 1.581, 95% CI 1.223–2.043; *p* < 0.001). (ii) Pathological conditions: bone fractures showed the strongest pronounced association with SADRs (OR 2.900, 95% CI 1.703–4.939; *p* < 0.001), followed by cerebral infarction (OR 2.658, 95% CI 1.827–3.866; *p* < 0.001), hemorrhagic disorder (OR 1.984, 95% CI 1.272–3.094; *p* = 0.003), cardiac insufficiency (OR 1.694, 95% CI 1.247–2.303; *p* = 0.001) and active malignancies (OR 1.386, 95% CI 1.016–1.890; *p* = 0.040). (iii) Drug exposure: Among anti-infective drugs, cefoperazone/sulbactam showed the highest association (OR 9.499, 95% CI 5.187–17.397; *p* < 0.001), exceeding antiviral medications (OR 5.484, 95% CI 2.597–11.578; *p* < 0.001), vancomycin (OR 5.021, 95% CI 2.560–9.848; *p* < 0.001), ceftriaxone (OR 4.259, 95% CI 2.029–8.939; *p* < 0.001), ceftazidime (OR 3.457, 95% CI 1.964–6.083; p < 0.001), antifungal agents (OR 2.922, 95% CI 1.390–6.142; *p* = 0.005) and carbapenems (OR 2.880, 95% CI 1.331–6.232; *p* = 0.007). Among other types of drugs, antiepileptics (OR 5.732, 95% CI 3.139–10.467; *p* < 0.001) was higher than that of proton pump inhibitors (OR 5.283, 95% CI 2.746–10.164; *p* < 0.001), diagnostic agents (OR 3.884, 95% CI 2.538–5.944; *p* < 0.001), cytotoxic antineoplastics (OR 2.497, 95% CI 1.618–3.853; *p* < 0.001), antithrombotic agents (OR 2.271, 95% CI 1.351–3.818; *p* < 0.001). (iv) IA administration (OR 2.768, 95% CI 1.126–6.809; *p* = 0.027) also had a significant risk.

### Comparison of SADRs prediction model by different ML

**FIGURE 2:**
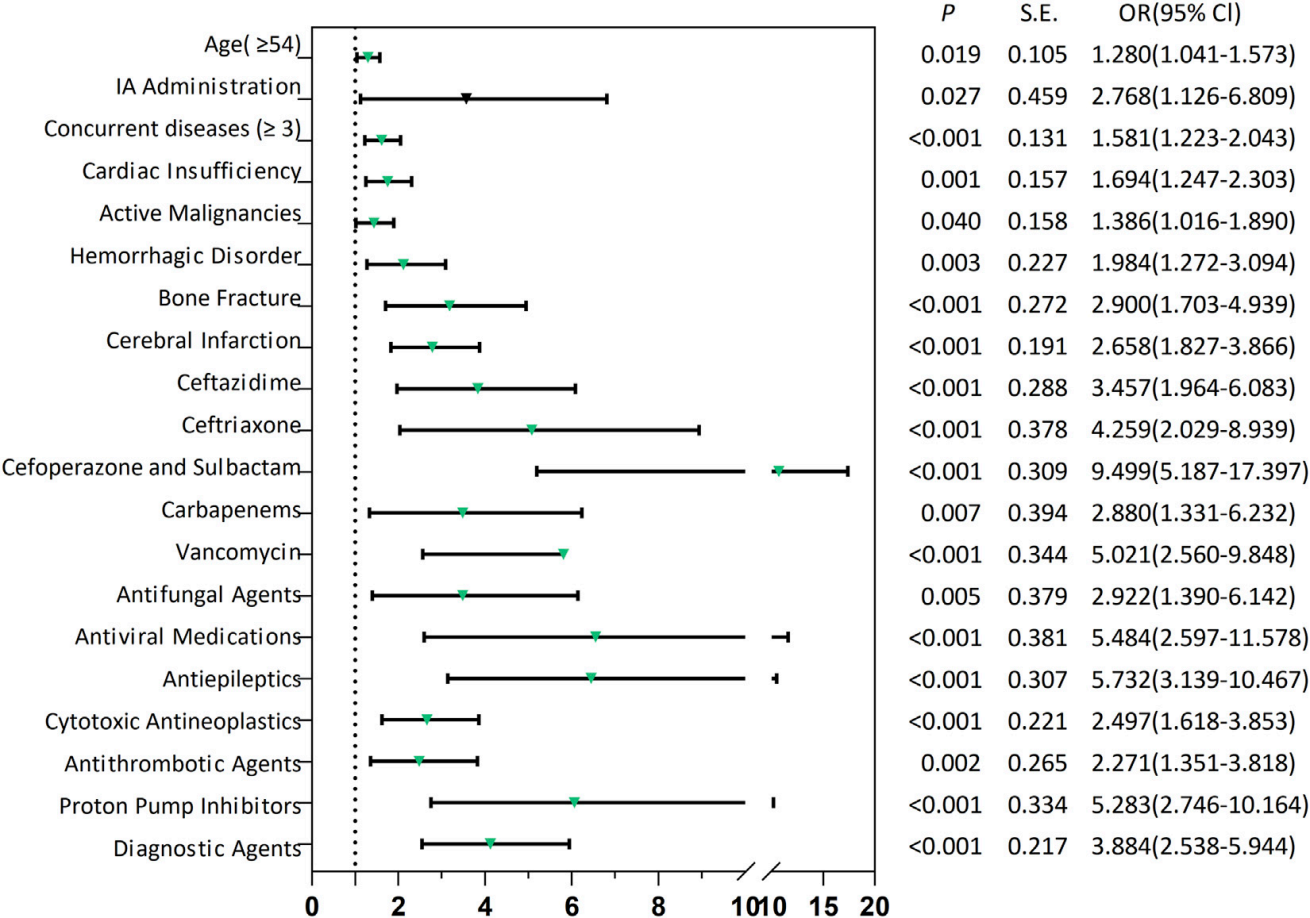
Forest plots of risk factors associated with SADRs. SADRs: serious adverse drug reactions; IA Administration: Intra-arterial Administration.

**FIGURE 3:**
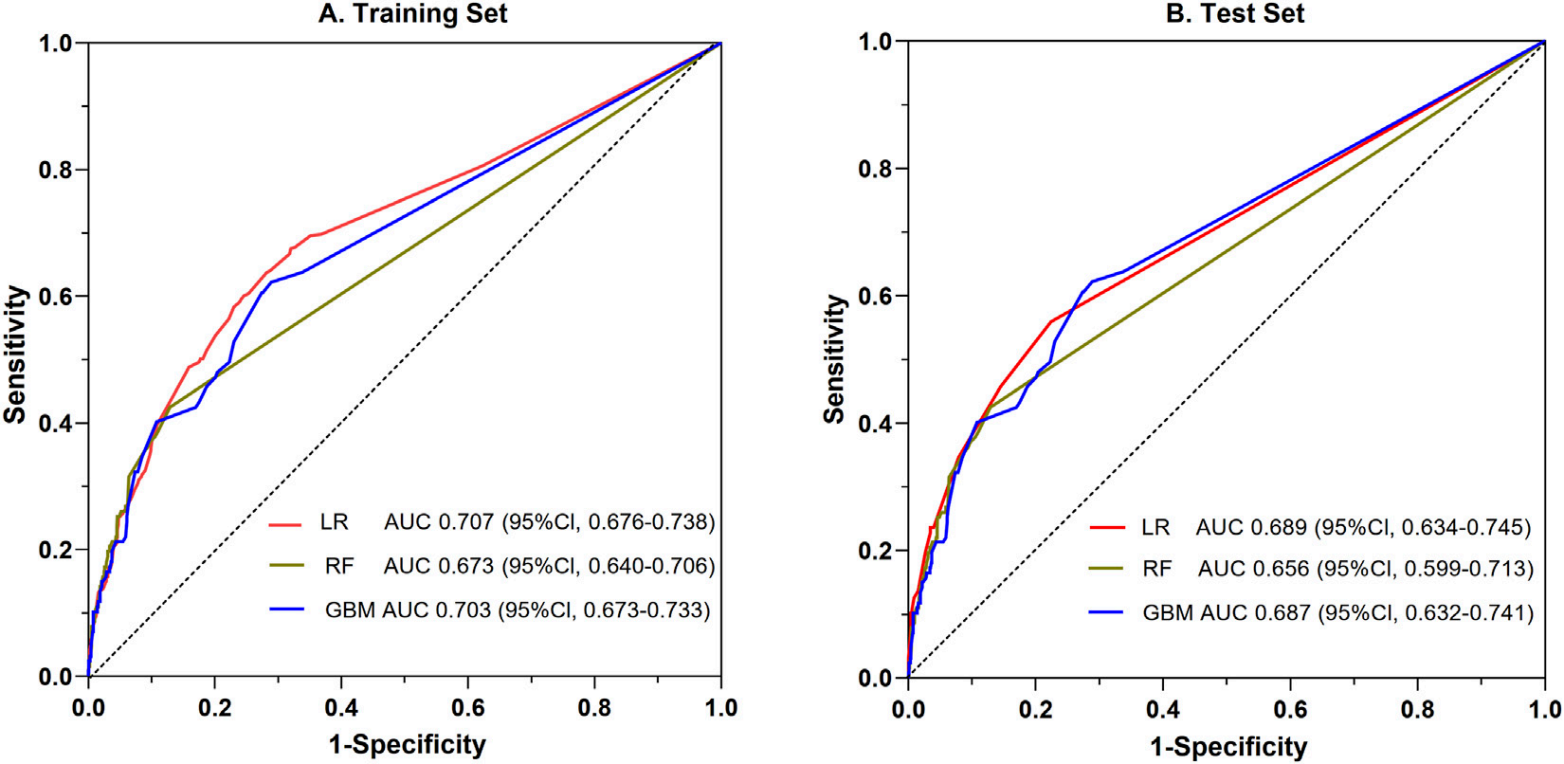
Comparative evaluation of ROC among the three ML models for the prediction of SADRs. ROC: the receiver operating characteristic curve; LR: logistic regression; RF: random forest; GBM: gradient boosting machine.

Utilizing the 20 variables selected (Figure 2), we employed three ML algorithms to construct an prediction model for SADRs. Following rigorous nested hyperparameter tuning, the possibility that the observed superiority arose from overfitting or stochastic parameter selection was effectively ruled out. As revealed in Figure 3A, AUC values of the LR, RF and GBM models were 0.707, 0.673, and 0.703 respectively based on the training set. Among the three models, LR showed the highest level of predictive accuracy (AUC = 0.707, 95% CI 0.676–0.738). The results of the test set were similar to the training sets (Figure 3B). The additional performance metrics of each model, including specificity, accuracy, sensitivity, F1-score and precision, are detailed in Supplementary File 2; Supplementary Tables S8, S9.

### Establishment of a nomogram model for SADRs

**FIGURE 4:**
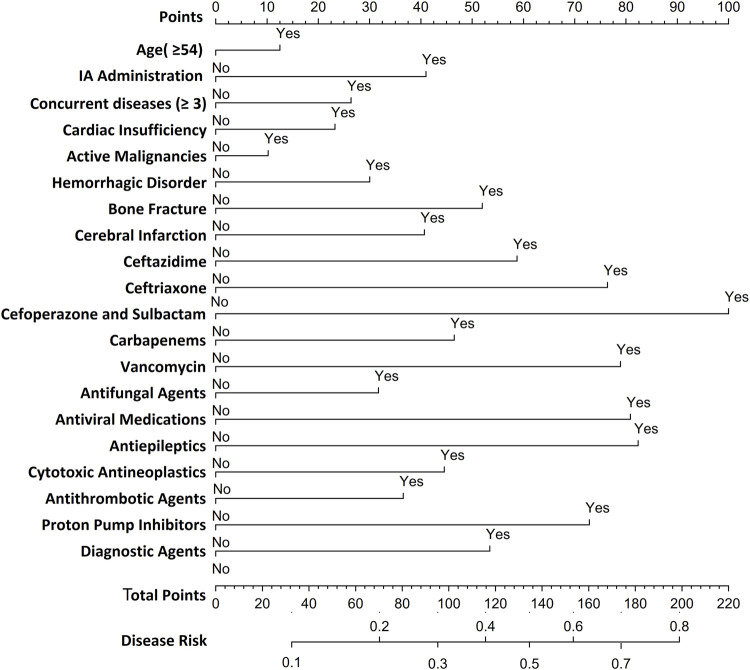
Nomogram for predicting SDARs. To estimate the probability of SADRs, mark patient values at each axis, draw a straight line perpendicular to the point axis, and sum the points for all variables. Next, mark the sum on the total point axis and draw a straight line perpendicular to the probability axis.

Utilizing LR-derived predictors, a prognostic nomogram was developed to visualize individual risk factors (Figure 4). This graphical model incorporated all significant covariates identified through multivariate LR, with weighted point allocations reflecting effect magnitudes as detailed in Figure 2. Each risk factor was assigned a specific score, and the cumulative scores of all risk factors can correspond to the predicted probability of SADRs occurrence.

### Evaluation of a nomogram model for predicting SADRs

**FIGURE 5:**
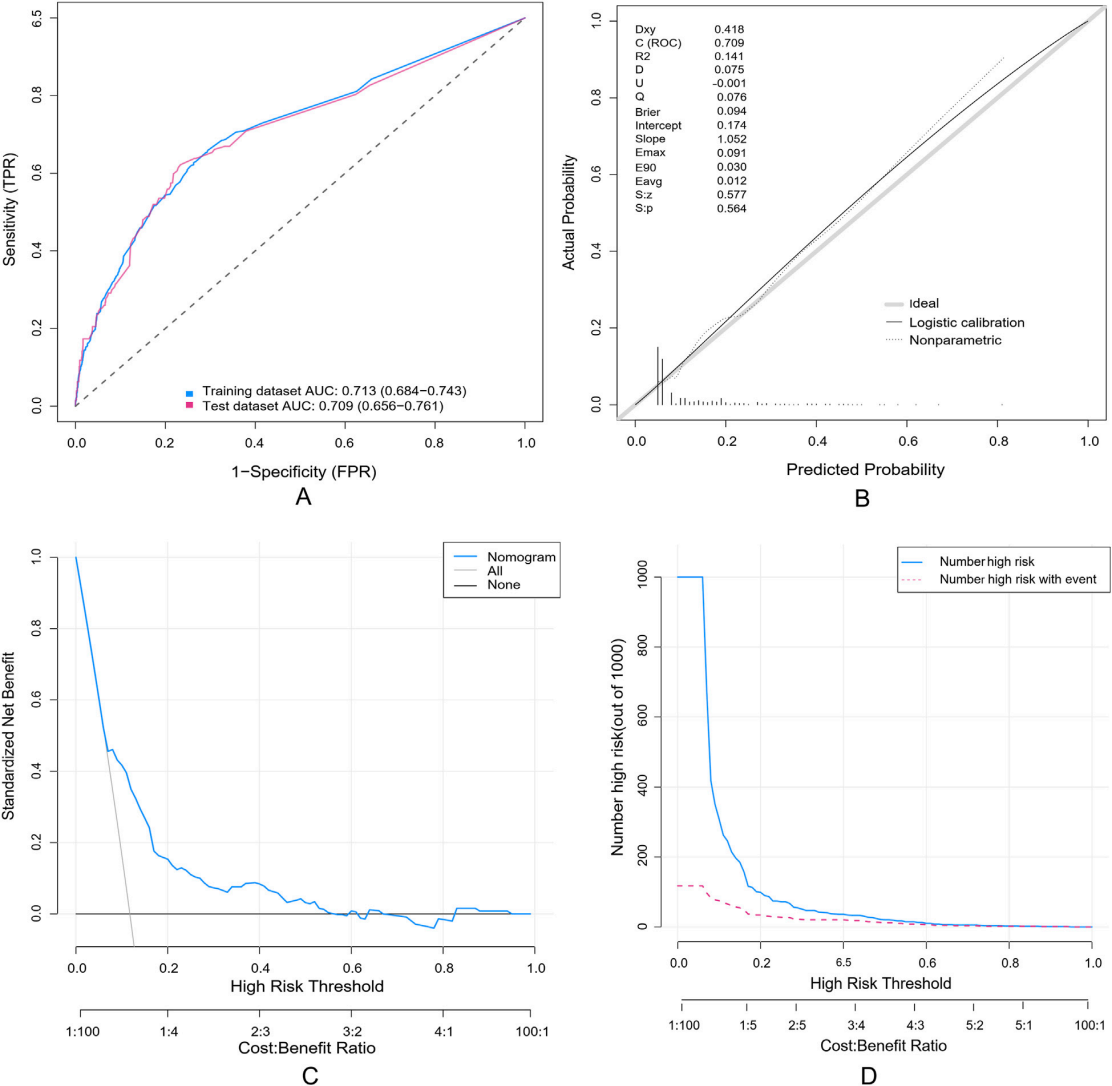
Evaluation of a nomogram model for predicting SADRs. **(A)** Receiver Operating Characteristic curve (ROC) of the training set and the test set. The area under the curve (AUC) of the purposed nomogram for predicting SADR in the training set and in the test set was 0.713 and 0.709, respectively. **(B)** The calilbration of the nomogram. The Calibration curve analysis of the nomogram in the test set. **(C)** The DCA of the nomogram for predicting SADR in the test set. The DCA curve of the nomogram. it was revealed that SADR prediction using the nomogram isaccompanied with a higher net benefit than using any single factor alone, as wel as by treating either no or all patients. **(D)** The CIC curve of the purposed nomogram in the test set. The red curve (the number of individuals at high risk) indicates the number of persons who are classified as positive (high risk) by the prediction model at each threshold probability; the blue curve (the number of individuals at high risk with outcomes) is the number of true positives at each threshold probability.

A random partitioning of the data set resulted in a 75% training set and a 25% test set. Discriminative performance evaluation demonstrated moderate predictive accuracy in the training set (Figure 5A), with an AUC of 0.713 and a bootstrap-corrected C-index of 0.714. Calibration accuracy, assessed via 1000-resample bootstrap validation, revealed strong agreement between predicted probabilities and observed outcomes (Hosmer-Lemeshow goodness-of-fit test: χ^2^ = 9.769, *p* = 0.369), as visualized in the calibration curve (Figure 5B). DCA quantified clinical translatability across a threshold probability range of 7%–56%. Within this range of clinical relevance, the nomogram demonstrated a higher net benefit compared to the “treat-all” or “treat-none” strategies (Figure 5C), with the optimal threshold probability resulting in a maximum absolute risk reduction of 32.5%. The CIC illustrated that with a 20% threshold, the model’s prediction of at-risk individuals greatly surpassed the real number (Figure 5D). When the threshold probability was above 55%, the predicted number of high-risk subjects (predicted positive cases by the scoring system) was nearly identical to the true high-risk cases, indicating the nomogram model’s significant predictive value for SADRs.

## Discussion

SADRs are critical concerns in pharmacovigilance, linked to prolonged hospitalization, higher medical costs, and adverse clinical outcomes. This retrospective study analyzed 4,333 ADRs, including 508 SADRs, aiming to identify SADR determinants. Three ML algorithms were developed for SADR prediction. The LR model outperformed RF and GBM. Subsequently, a nomogram was developed using the LR model.

It is important to highlight that in the present study, the continuous variable of age was dichotomized. The cut-off point of ≥54 years was determined based on the threshold corresponding to the optimal cut-off point in ROC analysis, rather than arbitrarily setting 60 years as the age limit for the elderly (Obuchowski and Bullen, 2018). This data-driven approach is consistent with the methodology employed in the pharmacovigilance study conducted by Han YZ et al., which identified 52 years as the optimal cut-off point for predicting ADRs of hepatotoxicity (Han et al., 2022). Consistent with the findings reported by Toni E et al., the present study corroborated the significant roles of age, comorbidities, and specific drug types in the occurrence of SADRs (Toni et al., 2024a; Toni et al., 2024b). For patients identified as high-risk, targeted preventive strategies can be implemented. By employing close monitoring or optimizing treatment plans, potential adverse reactions can be promptly detected and managed, thereby enhancing both the efficacy and safety of therapeutic interventions for these patients.

The application of ML techniques to predict SADRs is essential, particularly when conducted in accordance with relevant policies. This approach not only enhances the standardization and interpretability of research data but also promotes regulatory consistency and facilitates cross - departmental collaboration (Toni and Ayatollahi, 2025). ML, encompassing unsupervised learning, supervised learning, reinforcement learning, etc., has demonstrated notable advantages in ADR-related studies (Salas et al., 2022). However, differences in study population, data structure and confounding factors contribute to variations in performing of different ML algorithms for predicting ADRs (Deimazar and Sheikhtaheri, 2023). In the systematic report by Deimazar G et al., eight comparative studies on various ML algorithms for ADR prediction reported inconsistent results (Deimazar and Sheikhtaheri, 2023). In a study comparing three ML algorithms--LR, decision trees, and artificial neural networks-for predicting chemotherapy - induced ADRs, LR model had the highest AUC (0.67–0.83) for the six types of ADRs caused by chemotherapy drugs (On et al., 2022).

Following hyperparameter optimisation, the LR model exhibited the best discriminative performance among the three ML models we established, a finding that aligns with recent evidence (On et al., 2022). In a cross-sectional study to predict osteoporosis in older adults at high risk of cardiovascular disease, the LR model outperformed SVM, Random Forest, XGBoost, and Decision Tree models (Peng et al., 2025). Guo Y. et al. developed an ultrasound-based radiomics nomogram for identifying HER2 status in breast cancer patients, the LR model was found to perform the best on the validation set (Guo et al., 2022). Similarly, Xu R et al. reported that the LR model exhibited the highest discriminative power in identifying individuals with low bone mineral density using ML algorithms (Xu et al., 2024). Christodoulou E et al. compared the predictive capabilities of LR with other machine learning models, concluding that LR often performs comparably to or even outperforms ML algorithms in clinical prediction studies, particularly in datasets with limited sample sizes (Christodoulou et al., 2019). While ML algorithms hold theoretical advantages, especially in datasets with complex interactions or large sample sizes (Peng et al., 2025). In contrast, the LR model demonstrates strong reliability and applicability in clinical predictive analytics, with enhanced interpretability for binary outcomes (Dsouza et al., 2025). This highlights that LR and other ML models may be applicable in distinct scenarios: the LR model is preferable in studies with limited sample sizes, a small number of predictors, or low signal-to-noise ratio (SNR) data. Conversely, other ML models are more suitable for large datasets with numerous predictors, complex interactions/confounding factors, or high-SNR data (Christodoulou et al., 2019; Wei et al., 2024). We posit that even with hyperparameter tuning, the LR model may still exhibit advantages in datasets characterized by limited sample sizes and low signal-to-noise ratios. In future experiments, we recommend conducting a more extensive exploration of the performance of various models across different datasets, particularly under conditions of varying sample sizes and signal-to-noise ratios. Additionally, we suggest experimenting with a broader range of models and tuning methods to further validate the reliability of the aforementioned conclusion.

Nomograms have been identified as an efficient means of measuring ADRs risks in the past few years. Bai H et al. developed a nomogram for hospitalized adult patients to predict cefoperazone/sulbactam-induced hypoprothrombinemia, which demonstrated satisfactory predictive performance (Bai et al., 2023). Li P et al. constructed a nomogram to predict granisetron-associated arrhythmias, showing high discriminative and calibratioan capabilities (Li et al., 2024). Hong H et al. designed a predictive model with high efficacy for identifying vancomycin-induced acute kidney injury in overweight patients (Hong et al., 2024). Zhang Z et al. created a nomogram to forecast cutaneous adverse reactions induced by targeted cancer therapies and immunotherapy, offering critical insights for optimizing treatment efficacy and improving quality of life (Zhang Z. M. et al., 2025). However, to the best of our knowledge, no nomogram model specific to SADRs has been developed, particularly among large hospitalized populations using routinely collected clinical data.

Based on the LR model, we developed a nomogram to predict SADRs. This nomogram integrated multiple predictors as scaled axes aligned on a single plane, enabling clinicians to intuitively visualize individualized SADR probabilities. In clinical practice, physicians can utilize the nomogram model to assess the likelihood of SADR occurrence based on a patient’s specific risk factors. For instance, consider a 60-year-old male patient with more than three underlying diseases and heart insufficiency, who received treatment with cefoperazone/sulbactam for an infectious disease during hospitalization. According to the nomogram, his cumulative score for is 166, indicating a 60%–70% probability of SADR occurrence. Upon receiving this risk signal, physicians should implement stringent monitoring of the patient’s various clinical indicators following the administration of cefoperazone/sulbactam to preempt the occurrence of SADRs. Alternatively, physicians may consider substituting the anti-infective agent with piperacillin/tazobactam to mitigate the likelihood of SADR occurrence. The nomogram model we have developed is characterized by its simple structure and ease of understanding, thereby ensuring good interpretability. This feature not only facilitates the application and validation of the model across different settings but also provides convenience for future collaborative efforts among multiple institutions and departments. Therefore, this study offers theoretical value by enriching and expanding the methodological framework of SADR monitoring and pharmacovigilance, and serves as a reference for the development of personalized early-warning systems for SADRs.

The study presents multiple limitations. Firstly, it is a two-center retrospective cohort study. While it provided a preliminary pharmacovigilance approach, it was constrained by a small sample size and limited inclusion of variables, necessitating validation through larger, multicenter studies. Second, the study was restricted to the Han Chinese population, limiting its generalizability to other ethnicities. Third, the present study acknowledges several methodological limitations. Pharmacokinetic and pharmacogenomic parameters were not incorporated; future research could integrate these factors to develop a more comprehensive predictive model. Fourthly, despite our model demonstrating acceptable discrimination in internal validation (AUC = 0.713; C-index = 0.714), its performance remains at a moderate level. This suggests that while the nomogram holds value for signal detection within the existing data range, it is not yet robust enough to serve as an independent clinical decision-making tool. Further validation through multicenter, prospective cohort studies, or randomized clinical trials is necessary to comprehensively assess its calibration, clinical utility threshold, and cost-effectiveness, thereby delineating its true scope of application.

Future research on SADRs will be pursued along five complementary trajectories to strengthen surveillance and advance pharmacovigilance: (i) All variables in this study are derived from the mandatory reporting fields of the national “Drug Adverse Reaction Monitoring Specifications”. The two hospitals involved exhibit differences in demographics and drug catalogues, suggesting that the model has the potential for cross-regional migration. Subsequently, a prospective validation will be conducted, employing the same variable set and thresholds, and reporting the C-index, calibration slope, and decision curve to further evaluate the external validity. (ii) Employing ML models to perform sub-classification-based predictions of SADRs, thereby achieving a higher level of prognostic precision. (iii) Harnessing natural language processing for large-scale corpus analysis and integrating it with ML algorithms to shift SADR surveillance from retrospective tracing to prospective, real-time early warning (Le Glaz et al., 2021; Khanbhai et al., 2021). (iv) Embedding validated ML-SADR prediction modules into the Hospital Pharmacovigilance System to enable continuous, real-time monitoring and support proactive pharmacovigilance. (v) Collaborative research endeavors involving multiple stakeholders, including research institutions, pharmaceutical companies, and government agencies (Toni and Ayatollahi, 2025).

## Conclusion

The occurrence of SADRs is associated with multiple factors. This study identified key predictors of SADRs and construct a nomogram, which facilitated prophylactic surveillance of high-risk populations.

## Data Availability

The original contributions presented in the study are included in the article/Supplementary Material, further inquiries can be directed to the corresponding author.
