# Supplementary material for: Development and validation of a predictive nomogram for severe adverse drug reactions: a dual-center pharmacovigilance study
Source: Front Pharmacol. 2025 Nov 7;16:1669995. doi: 10.3389/fphar.2025.1669995 (PMC12634630; doi:10.3389/fphar.2025.1669995)
Supplement: Supplementary file 1 [file Supplementaryfile1.docx]

Supplementary file 1

Hyperparameter Tuning Documentation

For each algorithm, hyper-parameters were optimised by grid search within a 5-fold stratified nested cross-validation (CV) on the training set.

The inner loop identified the best combination from the following grids:

(1) *LR*: inverse-regularisation, *C* ∈ {0.001, 0.01, 0.1, 1, 10, 100};

(2) *RF*: *n_estimators* ∈ {100, 300, 500}, *max_depth* ∈ {3, 5, 7, None}; *max_features*∈ {auto, sqrt, log2, 0.5, 0.75, 10, 20};

(3)*GBM*: *learning_rate* ∈ {0.01, 0.05, 0.1}, *n_estimators* ∈ {100, 300, 500}, *max_depth* ∈ {3, 5, 7};

The outer loop estimated generalisation performance with ROC-AUC.

After nested CV, the best hyper-parameters were locked and the model was re-trained on the entire training set and evaluated once on the independent test set.

All procedures were implemented in R 3.5.1 using the caret package (v 6.0-81) with a fixed random seed (such as 12345 or 44).
