# Supplementary material for: Development and validation of a predictive nomogram for severe adverse drug reactions: a dual-center pharmacovigilance study
Source: Front Pharmacol. 2025 Nov 7;16:1669995. doi: 10.3389/fphar.2025.1669995 (PMC12634630; doi:10.3389/fphar.2025.1669995)
Supplement: Supplementary file 2 [file Supplementaryfile2.docx]

Supplementary file 2

# Supplementary Tables

**Table list**

Table **S1**. Organ-specific injury manifestations of SADRs.

Table **S2**. Comparisons between organs/systems involved in common ADRs and SADRs.

Table **S3**. Common ADRs and SADRs caused by different types of medicines.

Table **S4**. Univariate analysis of SADRs related to comorbidities

Table **S5**. Univariate analysis of SADRs related to medications

Table **S6**. The scores of the significant influencing factors of the multivariate logistic regression model and the collinearity diagnosis

Table **S7** Multivariate analysis of SADRs

Table **S8**. Predictive performance of four ML models on the training set

Table **S9**. Predictive performance of four ML models on the test set

Table **S1**. Organ-specific injury manifestations of SADRs

| **(1) Systemic organ injury**(152): rigors(61), fever(42), anaphylactic shock(21), hyperhidrosis(14), high fever(11), shock(5), fear of cold(4), syncope(3), allergic reaction(3), fatigue(3), infusion reaction(1), shivering(1), aconitine-like reaction(1), multiple organ failure(1), sense of impending death(1). |
| --- |
| **(2) Damage to the skin and its appendages**(181): pruritus(103), eruption(96), erythematous rash(34), papule(26), erythema(9), wheal(8), flush(5), urticaria(4), systemic redness(3), erythema multiforme(3), severe erythema multiforme-type drug eruption(2), exfoliative dermatitis(2), blister(2), drug eruption(2), rubella(2), localized skin reaction(2), skin desquamation(1), erosive balanoposthitis(1), epidermolysis bullosa type eruption(1), acute urticaria(1), contact dermatitis(1), skin necrosis(1). |
| **(3) Damage to the gastrointestinal tract(44)**: nausea(33), emesis(19), abdominal pain(6), diarrhoea(3), abdominal bloating(2), anorexia(2), decreased appetite(1). |
| **(4) Damage to the central and peripheral nervous systems**(68): vertigo(15), twitch(9), headache(6), ocalized numbness(6), limb tremors(5), coma(4), mental abnormality(4), loss of consciousness(4), pain(4), tremor of the limbs(3), consciousness disorder(3), twitching(3), delirium(3), somnolence(2), abnormal electroencephalogram(1), neurotoxicity(1), tremble(2), hallucination(1), panic(1), clouding of consciousness(2), restless(2), mania(1), irritable(1), panicoid reaction(1), prolonged emergence(1), language disorder(1), dizziness(1), agitation(1), epileptic seizure(1), myoclonus(1), lnumbness of the tongue(1). |
| **(5) Damage to the respiratory system**(63): dyspnea(30), chest tightness(27), difficulty breathing(13), tachypnea (7), dyspnea(3), worsening cough(2), wheezing(2), stridor(1). |
| **(6) Damage to the cardiovascular system**(69)  (i)Disorder of heart rate and rhythm(41): heart palpitations(16), palpitations(12), arrhythmia(7), tachycardia(4), cardiac discomfort(2), cardiac arrest(1).  (ii)Extracardiac vascular damage(6): cyanosis(5), venous thrombosis(1).  (iii)General damage to the cardiovascular system(22): decreased blood pressure(8), hypotension(5), hypertension(5), cold limbs(3), variations in blood pressure(1), amaurosis fugax(1). |
| **(7) Metabolic and nutritional disorders**(19): facial swelling(5), edema(5), eyelid edema(3), hypoglycemia(3), hypokalemia(2), hyponatremia(1), hyperuricemia(1), lactic acidosis(1). |
| **(8) Hepatobiliary dysfunction**(66): abnormal liver function(21), elevated transaminases(14), elevated liver enzymes(8), hepatic injury(6), elevated total bilirubin(2), abnormal liver enzymes(2), hepatitis(1), hepatocellular injury(1), gallbladder disease(1), elevated gamma-glutamyl transferase(1), acute liver failure(1). |
| **(9) Hematologic abnormalities**(52)  (i) Disorders of platelets, bleeding, and coagulation(30): thrombocytopenia(10), coagulopathy(3), prolonged prothrombin time(3), hematochezia(3), cerebral hemorrhage(3), gastrointestinal bleeding(2), hematoma(2), hyperemia of the conjunctiva(2), prolonged clotting time(1), non-specific bleeding(1), elevated INR(1), subcutaneous hemorrhage(1), hemolysis(1) .  (ii) Abnormalities of white blood cells and the reticuloendothelial system(19): leukopenia(8), myelosuppression(8), granulocytopenia(3).  (iii) Erythrocyte abnormalities(3): hemoglobin reduction(2), red blood cell reduction(1). |
| **(10) Urinary system injury**(18): renal dysfunction(6), urinary incontinence(5), hematuria(3), renal failure(3), acute kidney injury(1), urinary retention(1). |
| **(11)Visual damage**(4): blurred vision(2), abnormal vision(2).  **(12)Damage to the musculoskeletal system**(6): limb pain(3), rhabdomyolysis(1), myalgia(1), increased muscle tone(1).  **(13)Damage to the endocrine system**(1): hyperthyroidism(1).  **(14)Immune system damage**(2): Henoch-Schonlein purpura(2).  **(15)Others**(6): foaming at the mouth(2), scrotal swelling(1), lip disease(1), tongue swelling(1), numbness of the tongue(1), panniculiti(1). |

SADRs: Serious adverse drug reactions;

Table **S2**. Comparisons between organs/systems involved in common ADRs and SADRs

| **Organs/Systems** | **Common ADRs**  **(3825)** | **SADRs**  **(508)** | **X^2^** | ***p*** |
| --- | --- | --- | --- | --- |
| Systemic organ injury | 392(10.2%) | 152(29.9%) | 158.087 | **<0.001** |
| Damage to the skin and its appendages | 1610(42.1%) | 181(35.6%) | 7.721 | **0.005** |
| Damage to the gastrointestinal tract | 754(19.7%) | 44(8.7%) | 36.45 | **<0.001** |
| Damage to the central and peripheral nervous systems | 577(15.1%) | 68(13.4%) | 1.022 | 0.312 |
| Damage to the respiratory system | 452(11.8%) | 63(12.4%) | 0.146 | 0.702 |
| Damage to the cardiovascular system | 442(11.6%) | 69(13.6%) | 1.771 | 0.183 |
| Metabolic and nutritional disorders | 199(5.2%) | 19(3.7%) | 2.007 | 0.157 |
| Hepatobiliary dysfunction | 159(4.2%) | 66(13.0%) | 71.107 | **<0.001** |
| Hematologic abnormalities | 86(2.2%) | 52(10.2%) | 92.797 | **<0.001** |
| Urinary system injury | 19(0.5%) | 18(3.5%) | fisher | **<0.001** |
| Others | 134(3.5%) | 19(3.7%) | 0.074 | 0.786 |

SADRs: Serious adverse drug reactions

Table **S3**. Common ADRs and SADRs caused by different types of medicines

| **Drug categories** | **All ADRs** | **Common ADRs** | **SADRs^a^** | **Percentage**  **(%)** |
| --- | --- | --- | --- | --- |
| Anti-infectives | 1341 | 1180 | 161 | 12.0 |
| Traditional Chinese medicine preparations | 444 | 395 | 49 | 11.0 |
| Cardiovascular drugs | 415 | 387 | 28 | 6.7 |
| Central nervous system drugs | 322 | 264 | 58 | 18.0 |
| Anti-tumor drugs^b^ | 303 | 256 | 47 | 15.5 |
| Endocrine system drugs | 298 | 278 | 20 | 6.7 |
| Gastrointestinal drugs | 239 | 209 | 30 | 12.6 |
| Respiratory system drugs | 224 | 216 | 8 | 3.6 |
| Blood and hematopoietic system drugs | 201 | 167 | 34 | 16.9 |
| Vitamin, nutritional drugs and drugs for regulating water and electrolyte balance | 170 | 163 | 7 | 4.1 |
| Diagnostic agents | 152 | 108 | 44 | 28.9 |
| Reproductive system and urinary system drugs | 111 | 101 | 10 | 9.0 |
| Immune system agents | 82 | 72 | 10 | 12.2 |
| Other drugs | 31 | 29 | 2 | 6.5 |

a. SADRs: a. Serious adverse drug reactions;

b. Including cytotoxic antineoplastics, targeted anti-tumor therapies, immunotherapeutic agents, hormonal agents, and others.

Table **S4**. Univariate analysis of SADRs related to comorbidities

| **Factors** | **All ADRs**  **(N=4333)** | **ADRs**  **(n=3825)** | **SADRs**  **(n=508)** | **OR（95%CI）** | **P-value** |
| --- | --- | --- | --- | --- | --- |
| **Number of concurrent diseases** | | | | | |
| =2 | 908(21.0%) | 795(20.8%) | 113(22.2%) | 1.090(0.872-1.363) | 0.448 |
| ≥3 | 619(14.3%) | 487(12.7%) | 132(26.0%) | 2.406(1.931-2.998) | **<0.001** |
| **Comorbidities** | | | | | |
| Thyroid dysfunction | 82(1.9%) | 70(1.8%) | 12(2.4%) | 1.298(0.699-2.411) | 0.410 |
| Cardiac insufficiency | 434(10.0%) | 350(9.2%) | 84(16.5%) | 1.967(1.519-2.547) | **<0.001** |
| Hypertension | 302(7.0%) | 257(6.7%) | 45(8.9%) | 1.349(0.969-1.879) | 0.076 |
| Renal insufficiency | 184(4.2%) | 161(4.2%) | 23(4.5%) | 1.079(0.690-1.688) | 0.738 |
| Hepatic insufficiency | 223(5.1%) | 200(5.2%) | 23(4.5%) | 0.860(0.553-1.337) | 0.502 |
| Active malignancies | 650(15.0%) | 552(14.4%) | 98(19.3%) | 1.417(1.117-1.798) | **0.004** |
| Respiratory failure | 33(0.8%) | 27(0.7%) | 6(1.2%) | 1.681(0.691-4.092) | 0.252 |
| Chronic obstructive pulmonary disease | 107(2.5%) | 96(2.5%) | 11(2.2%) | 0.860(0.457-1.616) | 0.639 |
| Pulmonary infection | 191(4.4%) | 162(4.2%) | 29(5.7%) | 1.369(0.912-2.056) | 0.130 |
| Fever | 55(1.3%) | 50(1.3%) | 5(1.0%) | 0.750(0.298-1.891) | 0.543 |
| Hyperlipidemia | 39(0.9%) | 34(0.9%) | 5(1.0%) | 1.108(0.432-2.847) | 0.831 |
| Hemorrhagic disorder | 139(3.2%) | 103(2.7%) | 36(7.1%) | 2.756(1.863-4.076) | **<0.001** |
| Bone fractures | 95(2.2%) | 75(2.0%) | 20(3.9%) | 2.049(1.240-3.386) | **0.005** |
| Common cold | 86(2.0%) | 73(1.9%) | 13(2.6%) | 1.350(0.743-2.453) | 0.325 |
| Epilepsy | 27(0.6%) | 19(0.5%) | 8(1.6%) | 3.205(1.396-7.360) | **0.006** |
| Cerebral infarction | 199(4.6%) | 143(3.7%) | 56(11.0%) | 3.190(2.307-4.411) | **<0.001** |

SADRs: Serious adverse drug reactions.

Table **S5**. Univariate analysis of SADRs related to medications

| **Factors** | **All ADRs**  **(N=4333)** | **ADRs**  **(n=3825)** | **SADRs (n=508)** | **OR（95%CI）** | **P-value** |
| --- | --- | --- | --- | --- | --- |
| Drug combination | 1209(27.9%) | 1192(31.2%) | 17(3.3%) | 0.076(0.047-0.125) | **<0.001** |
| Ceftazidime | 78(1.8%) | 61(1.6%) | 17(3.3%) | 2.136(1.238-3.687) | **0.006** |
| Ceftriaxone | 40(0.9%) | 30(0.8%) | 10(2.0%) | 2.540(1.234-5.228) | **0.011** |
| Cefoperazone and sulbactam | 48(1.1%) | 25(0.7%) | 23(4.5%) | 7.208(4.060-12.799) | **<0.001** |
| Carbapenems | 40(0.9%) | 30(0.8%) | 10(2.0%) | 2.540(1.234-5.228) | **0.011** |
| Vancomycin | 44( 1.0%) | 30(0.8%) | 14(2.8%) | 3.585(1.888-6.808) | **<0.001** |
| Antifungal agents | 44(1.0%) | 34(0.9%) | 10(2.0%) | 2.239(1.099-4.560) | **0.026** |
| Antiviral medications | 37( 0.9%) | 27(0.7%) | 10(2.0%) | 2.825(1.359-5.870) | **0.005** |
| Traditional Chinese medicine preparations | 444(10.2%) | 395(10.3%) | 49(9.6%) | 0.927(0.678-1.267) | 0.634 |
| Antiepileptics | 54(1.2%) | 36(0.9%) | 18(3.5%) | 3.866(2.179-6.861) | **<0.001** |
| Cytotoxic antineoplastics | 231(5.3%) | 188(4.9%) | 43(8.5%) | 1.789(1.267-2.526) | **0.001** |
| Platinum-based drugs | 106(2.4%) | 88(2.3%) | 18(3.5%) | 1.560(0.931-2.613) | 0.091 |
| Antithrombotic agents | 94(2.2 %) | 71(1.9%) | 23(4.5%) | 2.507(1.552-4.051) | **<0.001** |
| Proton pump inhibitors | 44(1.0%) | 28(0.7%) | 16(3.1%) | 4.410(2.369-8.209) | **<0.001** |
| Diagnostic agents | 152(3.5%) | 108(2.8%) | 44(8.7%) | 3.264(2.269-4.695) | **<0.001** |

SADRs: Serious adverse drug reactions;

Table **S6.** The scores of the significant influencing factors of the multivariate logistic regression model and the collinearity diagnosis

| **Model** | **Score** | **Collinearity statistics** | |
| --- | --- | --- | --- |
|  |  | **Tolerance** | **VIF^a^** |
| Gender(Male) | 4.546 | 0.976 | 1.024 |
| Age( ≥54) | 24.732 | 0.914 | 1.094 |
| IA administration^b^ | 39.412 | 0.850 | 1.176 |
| Concurrent diseases( ≥ 3) | 64.317 | 0.772 | 1.295 |
| Cardiac insufficiency | 27.137 | 0.857 | 1.167 |
| Hypertension | 3.165 | 0.821 | 1.218 |
| Active malignancies | 8.307 | 0.720 | 1.390 |
| Hemorrhagic disorder | 27.882 | 0.938 | 1.066 |
| Bone fractures | 8.167 | 0.985 | 1.015 |
| Cerebral infarction | 54.316 | 0.877 | 1.141 |
| Ceftazidime | 7.784 | 0.993 | 1.007 |
| Ceftriaxone | 6.876 | 0.991 | 1.009 |
| Cefoperazone and sulbactam | 61.433 | 0.988 | 1.012 |
| Carbapenems | 6.876 | 0.989 | 1.011 |
| Vancomycin | 17.342 | 0.981 | 1.020 |
| Antifungal agents | 5.2 | 0.985 | 1.015 |
| Antiviral medications | 8.444 | 0.994 | 1.006 |
| Antiepileptics | 24.672 | 0.985 | 1.015 |
| Cytotoxic antineoplastics | 11.195 | 0.733 | 1.365 |
| Antithrombotic agents | 15.078 | 0.932 | 1.073 |
| Proton pump inhibitors | 26.076 | 0.983 | 1.017 |
| Diagnostic agents | 45.151 | 0.854 | 1.171 |

a. VIF: Variance inflation factor ; b. IA Administration: Intra-arterial Administration

Table **S7** Multivariate analysis of SADRs

| **Demographics and clinical characteristics** | **B** | **S.E.** | **Wald** | **df** | ***p*** | **Exp(B)** | **95% EXP(B)** | |
| --- | --- | --- | --- | --- | --- | --- | --- | --- |
| Age( ≥54) | 0.247 | 0.105 | 5.477 | 1 | 0.019 | 1.280 | 1.041 | 1.573 |
| IA administration | 1.018 | 0.459 | 4.918 | 1 | 0.027 | 2.768 | 1.126 | 6.809 |
| Concurrent diseases(≥3) | 0.458 | 0.131 | 12.218 | 1 | <0.001 | 1.581 | 1.223 | 2.043 |
| Cardiac insufficiency | 0.527 | 0.157 | 11.348 | 1 | 0.001 | 1.694 | 1.247 | 2.303 |
| Active malignancies | 0.326 | 0.158 | 4.237 | 1 | 0.04 | 1.386 | 1.016 | 1.890 |
| Hemorrhagic disorder | 0.685 | 0.227 | 9.136 | 1 | 0.003 | 1.984 | 1.272 | 3.094 |
| Bone fractures | 1.065 | 0.272 | 15.356 | 1 | <0.001 | 2.900 | 1.703 | 4.939 |
| Cerebral infarction | 0.978 | 0.191 | 26.137 | 1 | <0.001 | 2.658 | 1.827 | 3.866 |
| Ceftazidime | 1.24 | 0.288 | 18.503 | 1 | <0.001 | 3.457 | 1.964 | 6.083 |
| Ceftriaxone | 1.449 | 0.378 | 14.676 | 1 | <0.001 | 4.259 | 2.029 | 8.939 |
| Cefoperazone and sulbactam | 2.251 | 0.309 | 53.175 | 1 | <0.001 | 9.499 | 5.187 | 17.397 |
| Carbapenems | 1.058 | 0.394 | 7.216 | 1 | 0.007 | 2.88 | 1.331 | 6.232 |
| Vancomycin | 1.614 | 0.344 | 22.036 | 1 | <0.001 | 5.021 | 2.560 | 9.848 |
| Antifungal agents | 1.072 | 0.379 | 8 | 1 | 0.005 | 2.922 | 1.390 | 6.142 |
| Antiviral medications | 1.702 | 0.381 | 19.919 | 1 | <0.001 | 5.484 | 2.597 | 11.578 |
| Antiepileptics | 1.746 | 0.307 | 32.303 | 1 | <0.001 | 5.732 | 3.139 | 10.467 |
| Cytotoxic antineoplastics | 0.915 | 0.221 | 17.08 | 1 | <0.001 | 2.497 | 1.618 | 3.853 |
| Antithrombotic agents | 0.82 | 0.265 | 9.586 | 1 | 0.002 | 2.271 | 1.351 | 3.818 |
| Proton pump inhibitors | 1.665 | 0.334 | 24.862 | 1 | <0.001 | 5.283 | 2.746 | 10.164 |
| Diagnostic agents | 1.357 | 0.217 | 39.063 | 1 | <0.001 | 3.884 | 2.538 | 5.944 |
| Constant | -2.893 | 0.092 | 984.676 | 1 | <0.001 | 0.055 |  |  |

SADRs: Serious adverse drug reactions; IA Administration: Intra-arterial Administration.

Table **S8**. Predictive performance of four ML^a^ models on the training set

| **Model** | **Area under curve b(AUC%)** | **Accuracy(%)** | **Sensitivity(%)** | **Specificity(%)** | **Precision** | **F1-score** |
| --- | --- | --- | --- | --- | --- | --- |
| LRc | 0.707 | 0.7086 | 0.6380 | 0.7180 | 0.2310 | 0.3392 |
| RFd | 0.673 | 0.8086 | 0.4590 | 0.8550 | 0.2960 | 0.3599 |
| GBMe | 0.703 | 0.6896 | 0.6640 | 0.6930 | 0.2232 | 0.3340 |

a.ML: Machine learning; b.AUC: area under the curve; c.LR: logistic regression; d.RF: random forest; e.GBM: gradient boosting machines

Table **S9**. Predictive performance of four ML^a^ models on the test set

| **Model** | **Area under curve ^b^(AUC%)** | **Accuracy(%)** | **Sensitivity(%)** | **Specificity(%)** | **Precision** | **F1-score** |
| --- | --- | --- | --- | --- | --- | --- |
| LRc | 0.689 | 0.7506 | 0.5590 | 0.7760 | 0.2489 | 0.3445 |
| RFd | 0.656 | 0.8187 | 0.4250 | 0.8710 | 0.3044 | 0.3547 |
| GBMe | 0.687 | 0.7006 | 0.6220 | 0.7110 | 0.2223 | 0.3275 |

a.ML: Machine learning; b.AUC: area under the curve; c.LR: logistic regression; d.RF: random forest; e.GBM: gradient boosting machines
